# The Danish Infection Cohort: a resource for population based infectious disease epidemiology

**DOI:** 10.1007/s10654-026-01410-5

**Published:** 2026-05-27

**Authors:** Noelle M. Cocoros, Eyal Oren, Bianka Darvalics, Kasper Mortensen, Heidi Amalie Rosendahl Jensen, Marie Holm Eliasen, Lars Pedersen, Henrik Toft Sørensen

**Affiliations:** 1 Department of Clinical Epidemiology, Center for Population Medicine, Aarhus University Hospital and Aarhus University, Olof Palmes Allé 43-45, Aarhus, 8200 Denmark; 2 Department of Population Medicine, Harvard Medical School, Harvard Pilgrim Health Care Institute, Boston, MA USA; 3 Division of Epidemiology and Biostatistics, School of Public Health, San Diego State University, San Diego, CA USA; 4 National Institute of Public Health, University of Southern Denmark, Copenhagen, Denmark; 5 Center for Clinical Research and Prevention, Copenhagen University Hospital – Bispebjerg and Frederiksberg, Frederiksberg, Denmark

**Keywords:** Infectious disease, Health survey, Epidemiology

## Abstract

Infections are a major cause of morbidity and mortality, but many aspects of the epidemiology of infections are unknown or unclear because key data are lacking. We created The Danish Infection Cohort by linking Danish National Health Survey data (including self-reported behavioral, health, and other information) to data on diagnoses and medications in Danish national health registries. We included individuals ≥ 18 years of age in surveys from 2010, 2013, 2017, and 2021. We identified evidence of infection in the 365 days post-survey by using two definitions: hospital-diagnosed infections, via primary or secondary discharge diagnoses in the Danish National Patient Registry (containing all Danish hospital-based outpatient, emergency, and inpatient encounters); and community-treated infections, via systemic anti-infective prescription redemptions at community pharmacies in the Danish National Prescription Registry. Overall, among 609,224 individuals, 196,980 (32.3%) had one or more infections within 365 days post-survey: 11,850 hospital-diagnosed and 185,130 community-treated. Because more than one infection occurred in some individuals, the total number of infections was 315,689: 25,385 hospital-diagnosed and 290,304 community-treated. The most common diagnoses were respiratory infections (34.9%), and the most common anti-infectives were antibiotics (80.6%). This cohort, with nearly 200,000 individuals with infections, and more than 315,000 infections overall, includes data on smoking, weight, physical activity, diet, alcohol use, and other factors, enabling a wide range of investigations including infectious disease patterns and etiologies over time.

**Supplementary Information:**

The online version contains supplementary material available at 10.1007/s10654-026-01410-5.

## Introduction

Infectious diseases are a major cause of morbidity and mortality globally [1]. Although infectious disease epidemiology is a mature field, substantial knowledge gaps persist. The relation between infections and factors such as health behavior (e.g., smoking and physical activity) [2] and weight status [3]are well recognized, yet studies examining infection risk and severity have been limited by a lack of availability of data on these factors. In addition, data on socioeconomic position, particularly at the individual level, are often unavailable. Even in Denmark and other Scandinavian countries whose national health registries contain extensive individual-level data [4, 5], information on weight and smoking is typically limited to diagnosis codes and prescriptions, thus leading to substantial under ascertainment and misclassification [6].

Herein, we describe The Danish Infection Cohort, which was designed to address key current and future knowledge gaps in the field of infectious disease epidemiology. To date, this cohort is comprised of individuals with evidence of a wide range of infections from 2010 to 2022. It was developed by linking respondents from the Danish National Health Survey, which collects self-reported information on lifestyle, behavioral, health, and other information, to individual-level information on diagnoses and treatments in the national Danish health registries. As described below, this cohort encompasses individuals with a broad range of infections, which is unique based on our review of the literature. Individual-level data on factors important to, but typically not available for, epidemiologic studies of infectious disease, combined with the longitudinal nature of the national registries also make this cohort distinct and well poised to answer a range of research questions.

## Methods

Denmark has ~ 6 million inhabitants and a universal, government-funded welfare system that ensures free and equal access to medical care based on residency [7, 8]. At birth or immigration, a unique identifier is assigned to each resident that enables linkage of individual-level data across all Danish registries.

### Data sources

Four data sources were used to create the cohort. The Danish Civil Registration System, established in April 1968, is an administrative population registry containing information on all people residing in Denmark [9]. The registry provides near-complete longitudinal data on emigration and death, and updates migration status and vital status daily.

The Danish National Health Survey has been conducted in 2010, 2013, 2017, 2021, 2023, and 2025, to date (although necessary data from surveys after 2021 were not available at the time of this analysis; www.danskernessundhed.dk). The overall aim of the survey is to describe the status and trends in health, health determinants, and morbidity in the adult population in Denmark. For each survey year, individuals ≥ 16 years of age are randomly selected from five mutually exclusive regional samples and one national sample. An introduction letter was sent to all selected individuals: in 2010 and 2013, hardcopy letters were sent by postal service; from 2017 on, letters were sent electronically by Danish Digital Post for those who were registered with the service (the majority) and by hardcopy letter for those who were not [10]. The survey is administered through identical paper and web-based questionnaires, depending on individual preference, and contains > 50 questions including demographics, lifestyle, behavioral, physical health, and mental health factors [10]. Survey topics have been consistent throughout the years, and most questions have been identical or similar at each survey implementation. Questions pertain to specific health conditions (e.g., asthma or arthritis), general health, stress, occurrence and effects of pain, physical symptoms, emotional health in daily activities, alcohol consumption, diet, physical activity, cigarette smoking, height, weight, educational level, employment status, social support, and health care engagement. See Supplemental Table 1 for information on survey participation by sex and age for the survey years included in the cohort [10, 11].

The Danish National Patient Registry contains all nonpsychiatric hospital-based diagnoses since 1977, and has included psychiatric inpatient, emergency, and hospital-based outpatient specialty clinic visits since 1995 [4]. For each encounter, a primary diagnosis code from the International Classification of Diseases (ICD) is recorded together with optional secondary diagnoses.

The Danish National Prescription Registry contains information on redeemed/dispensed prescriptions from community pharmacies since 1995 [5]. The core data elements in the registry include the specific medication dispensed, identified via Anatomical Therapeutic Chemical (ATC) classification system codes. The indication for the medication is also included as a variable [12, 13]. Over-the-counter medications are not included in the registry. However, systemic anti-infectives are available only by prescription in Denmark.

### Study population

We identified all individuals ≥ 18 years of age who responded to the National Health Survey from 2010 to 2021. For those who participated in more than one survey, we included data from only their first survey so we could report characteristics on the individual-level. Because the actual date of survey completion is unavailable, we used May 1 as the index date (day 0) for each survey year, as the survey is routinely administered between around the end of January and the beginning of May [8]. To ensure an adequate lookback window, we required individuals born outside of Denmark to have immigrated at least 5 years before their index dates.

For each individual, we assessed evidence of infection in the 365 days after the index date. We defined infections as hospital-diagnosed or community-treated. As our intention was to identify a range of infections for future studies, infections could be incident or prevalent (i.e., we did not apply a washout criterion). Hospital-diagnosed infections were identified according to a primary or secondary discharge diagnosis in the National Patient Registry according to ICD 10th Revision, Clinical Modification (ICD-10-CM) codes, including all care settings (outpatient, emergency, or inpatient). Diagnosis code lists were developed by the project team by using the Danish Health Data Agency browser (SKS, v4.06) and were reviewed at the individual code level. Community-treated infections were identified according to redemption at a community pharmacy of a systemic anti-infective prescription written by either a primary care or hospital-based physician in the National Prescription Registry, according to ATC codes. The ATC codes were restricted to systemic medications and were informed by Kristensen and colleagues [15] and reviewed by the project team. Each infection type was defined according to the presence of a single diagnosis code or prescription of interest (ICD-10-CM and ATC codes are in Supplemental Tables 2 and 3, respectively). We excluded prescriptions for which the “indication for use” variable suggested prophylactic purposes (e.g., malaria prophylaxis or prophylaxis for surgery) or purposes not related to infections (e.g., acne or rosacea).

Individuals were followed from May 1 of the survey year until first infection, emigration, death, or 365 days, whichever occurred first.

### Analyses

We assessed infections on the individual level and the infection level. For analyses on the individual level, we counted the first infection in the year after the survey response for hospital-diagnosed and community-treated infections separately. For analyses at the infection level, because each individual could contribute more than one infection during follow-up, the following rules were applied: the hospital-diagnosed infection was counted if a community-treated infection was registered within 30 days; the primary diagnosis was counted if multiple infection diagnoses were present in the same admission/visit; for individuals with more than one hospital diagnosis, or a prescription dispensed on different days within 30 days, only the first was counted; for individuals with more than one community-based anti-infective prescription dispensed on the same date, a hierarchy was applied (antiviral, then antibiotic, then antifungal) to count only one prescription.

In the survey data, we examined self-reported information by infection type. We assessed the presence of selected comorbidities from the National Patient Registry using a lookback window of 5 years before May 1 of the survey year (since some conditions can resolve over time, we used a 5-year lookback, but future work may use all available history [16]). We also assessed the use of selected medications in the 365 days before May 1 from the National Prescription Registry defined as a single dispensed prescription. ICD-10-CM and ATC codes for baseline covariates are available upon request. We examined the completeness of the “indication for use” variable included in the National Prescription Registry. From the National Health Survey we assessed cohabitation/marital status, educational level, self-rated health, body mass index (based on self-reported weight and height), and mental illness. Other self-reported information is also available from the survey, as reported by Christensen and colleagues [10]. Country of origin was identified in the Civil Registration System.

The underlying data from the Danish National Health Survey and the national registries are only accessible with appropriate permissions. Data cannot be made publicly available. Researchers who would like to collaborate with the Department of Clinical Epidemiology can contact dce@clin.au.dk. Details regarding the collaboration will be based on several factors. Alternatively, researchers can independently seek access from the Danish authorities. Data are available through application to the Danish health authorities (Statistics Denmark [www.dst.dk, dst@dst.dk] and the Danish Health Data Authority [www.sundhedsdatastyrelsen.dk, kontakt@sundhedsdata.dk]). Information regarding access to the National Health Survey data can be found here: www.danskernessundhed.dk/Dataudlevering.html.

All analyses were descriptive in nature and were conducted in SAS version 9.4. Researchers who are interested in the analytic code used to create the cohort can contact the Department of Clinical Epidemiology (dce@clin.au.dk).

This study is reported according to the Strengthening the Reporting of Observational Studies in Epidemiology (STROBE) guideline.

## Results

**Fig. 1:**
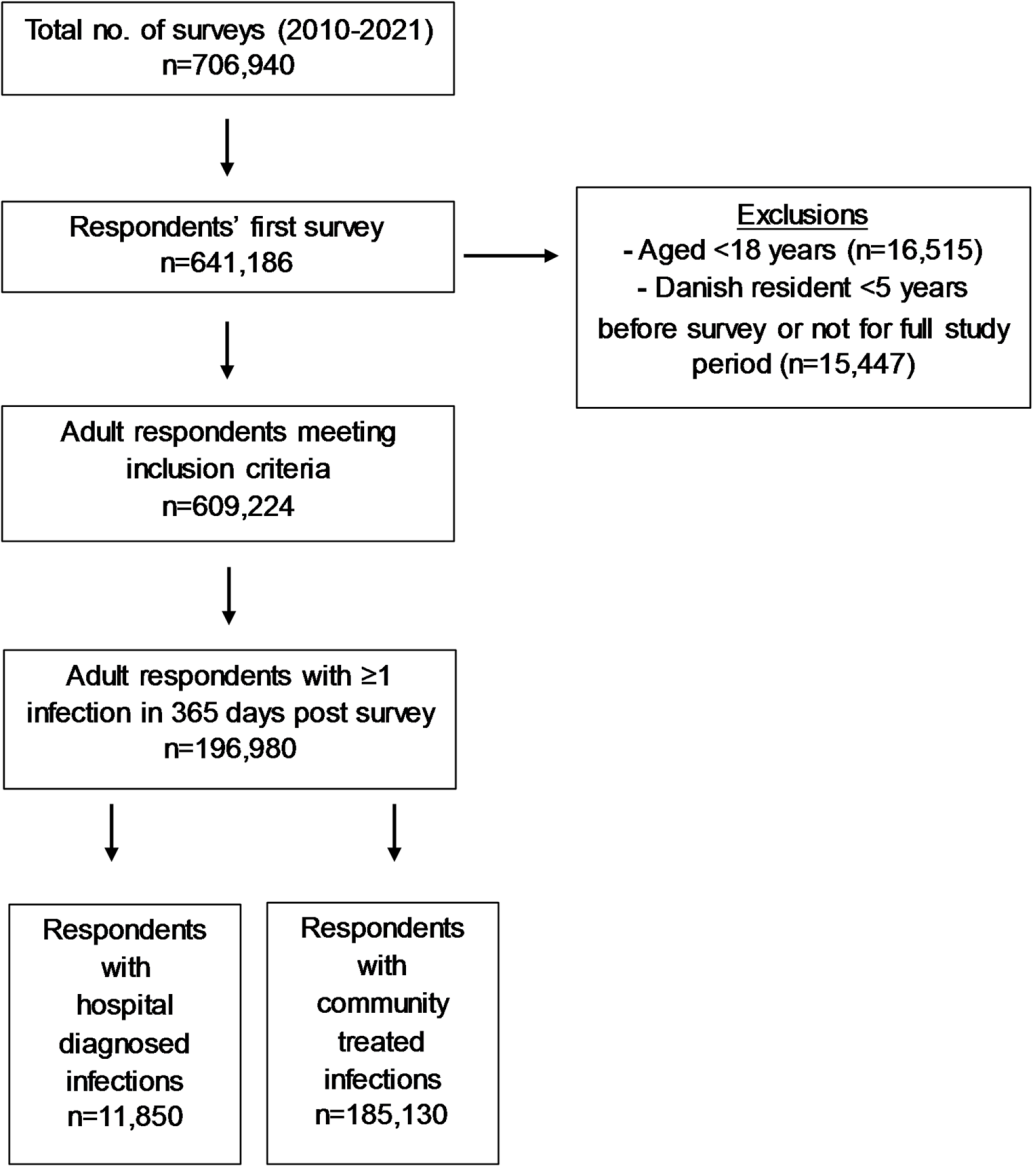
Flowchart of creation of The Danish infection cohort

The creation of the study cohort is depicted in Fig. 1. A total of 706,940 individuals completed the surveys across all years combined. When inclusion was restricted to those ≥ 18 years of age who were residing in Denmark for ≥ 5 years before the survey and through the study period, and to the first survey for individuals who participated in multiple surveys, a total of 609,224 remained. Among those individuals, 196,980 (32.3%) had at least one infection of either type in the 365 days after the survey response index date. When inclusion was restricted to the first infection during follow-up, 11,850 individuals had a hospital-diagnosed infection, and 185,130 individuals had a community-treated infection. The number and proportion of individuals with a hospital-diagnosed infection increased over time, from 19.7% among those in the 2010 survey to 31.9% among those in the 2021 survey (Table 1). In contrast, the number and proportion with a community-treated infection decreased over time, from 32.0% among those in the 2010 survey to 20.1% among those in the 2021 survey.

**Table 1:** Selected characteristics of The Danish Infection Cohort

	Eligible survey respondents		Those with a hospital-diagnosed infection		Those with a community-treated infection	
	No.	%	No.	%	No.	%
Total	609,224	100%	11,850	100%	185,130	100%
Female sex	329,085	54.0%	6,118	51.6%	117,599	63.5%
Median age in years (IQ range)	54.0 (40.0–67.0.0.0)		61.0 (41.0–74.0.0.0)		56.0 (40.0–68.0.0.0)	
*Age group (years)*						
18–24	49,970	8.2%	929	7.8%	14,578	7.9%
25–49	202,173	33.2%	3,098	26.1%	58,222	31.4%
50–69	238,385	39.1%	3,728	31.5%	70,071	37.8%
≥70	118,696	19.5%	4,095	34.6%	42,259	22.8%
*Year of survey*						
2010	169,524	27.8%	2,331	19.7%	59,329	32.0%
2013	140,393	23.0%	2,197	18.5%	44,440	24.0%
2017	152,947	25.1%	3,547	29.9%	44,076	23.8%
2021	146,360	24.0%	3,775	31.9%	37,285	20.1%
*Cohabitation/marital status* ^1^						
Married/living with partner	427,347	70.1%	7,549	63.7%	128,168	69.1%
Alone/divorced/widow	174,729	28.7%	4,106	34.7%	54,557	29.4%
Missing	7,148	1.2%	188	1.6%	2,739	1.5%
Denmark is country of origin^2^	572,369	94.0%	10,935	92.3%	174,880	94.5%
*Highest education level* ^1^						
Under education	32,470	5.3%	551	4.6%	9,406	5.1%
Secondary compulsory	268,390	44.1%	5,232	44.2%	81,846	44.2%
Vocational/high school	172,125	28.3%	2,916	24.6%	53,001	28.6%
Higher education	62,324	10.2%	1,154	9.7%	17,200	9.3%
Other education	26,932	4.4%	653	5.5%	8,450	4.6%
Missing	46,983	7.7%	1,344	11.3%	15,227	8.2%
*Self-rated health* ^1^						
Excellent	61,197	10.0%	764	6.4%	14,421	7.8%
Very good	212,139	34.8%	2,833	23.9%	56,430	30.5%
Good/well	234,228	38.4%	4,475	37.8%	72,651	39.2%
Less good/bad	95,690	15.7%	3,601	30.4%	39,425	21.3%
Missing	5,970	1.0%	177	1.5%	2,203	1.2%
*Smoking status* ^1^						
Never smoker	273,312	44.9%	4,456	37.6%	75,966	41.0%
Former	192,619	31.6%	4,205	35.5%	62,595	33.8%
Current	120,509	19.8%	2,575	21.7%	39,659	21.4%
Missing	22,784	3.7%	614	5.2%	6,910	3.7%
*Body mass index*^1^ (kg/m^2^)						
Underweight (<18.5)	11,359	1.9%	369	3.1%	3,892	2.1%
Normal weight (18.5 - <25)	263,260	43.2%	4,758	40.2%	79,115	42.7%
Overweight (25 - <30)	205,005	33.7%	3,691	31.1%	59,997	32.4%
Obesity (≥30)	96,229	15.8%	2,146	18.1%	32,011	17.3%
Missing	33,371	5.5%	886	7.5%	10,115	5.5%
*Mental illness lasting <6 mo* ^1^						
Yes	41,006	6.7%	1,085	9.2%	13,894	7.5%
No	363,443	59.7%	7,372	62.2%	100,505	54.3%
Missing	204,775	33.6%	3,393	28.6%	70,731	38.2%
*Mental illness lasting ≥6 mo* ^1^						
Yes	43,660	7.2%	1,164	9.8%	14,606	7.9%
No	363,061	59.6%	7,363	62.1%	100,523	54.3%
Missing	202,503	33.2%	3,323	28.0%	70,001	37.8%
*Diagnosed health conditions in 5 yrs prior to survey* ^3^						
Stroke	3,295	0.5%	403	3.4%	2,892	1.6%
Ischemic heart disease	9,439	1.5%	868	7.3%	8,563	4.6%
Chronic obstructive pulmonary disease	6,023	1.0%	691	5.8%	5,332	2.9%
Other chronic pulmonary disease (including asthma)	6,139	1.0%	551	4.6%	5,588	3.0%
Diabetes (excluding type 1)	7,941	1.3%	843	7.1%	7,098	3.8%
Type 1 diabetes	2,674	0.4%	257	2.2%	2,417	1.3%
Congestive heart failure	3,615	0.6%	500	4.2%	3,115	1.7%
Hypertension	18,981	3.1%	1,764	14.9%	17,217	9.3%
Atrial fibrillation and flutter	7,592	1.2%	866	7.3%	6,726	3.6%
Peripheral vascular disease	4,096	0.7%	414	3.5%	3,682	2.0%
Cerebrovascular disease	6,205	1.0%	692	5.8%	5,513	3.0%
Dementia	958	0.2%	117	1.0%	841	0.5%
Psychiatric disorder	10,085	1.7%	862	7.3%	9,223	5.0%
Kidney disease	3,078	0.5%	496	4.2%	2,582	1.4%
Liver disease	1,230	0.2%	164	1.4%	1,066	0.6%
Cancer (excluding non-melanoma skin cancer)	11,668	1.9%	1,253	10.6%	10,415	5.6%
*Charlson comorbidity index* ^3^						
0	544,509	89.4%	6,429	54.3%	126,956	68.6%
1–2	48,613	8.0%	3,520	29.7%	44,268	23.9%
≥3	16,102	2.6%	1,901	16.0%	13,906	7.5%
*Medication use in 365 days prior to survey* ^4^						
Immunosuppressants	2,586	0.4%	180	1.5%	2,406	1.3%
Oral anticoagulants	9,682	1.6%	1,074	9.1%	8,608	4.6%
Beta blockers	23,697	3.9%	2,018	17.0%	21,679	11.7%
Calcium channel blockers	24,678	4.1%	1,938	16.4%	22,740	12.3%
Angiotensin-converting enzyme inhibitors	23,145	3.8%	1,714	14.5%	21,431	11.6%
Angiotensin receptor blockers	21,755	3.6%	1,576	13.3%	20,179	10.9%
Psychoactive medications	38,308	6.3%	2,617	22.1%	35,691	19.3%
Systemic glucocorticoids	11,983	2.0%	949	8.0%	11,033	6.0%
Statins	38,544	6.3%	2,780	23.5%	35,764	19.3%
Non-steroidal anti-inflammatory drugs	41,825	6.9%	2,150	18.1%	39,675	21.4%
*Mortality* ^2^						
Died within 1 year of survey	5,582	0.9%	1,006	8.5%	2,554	1.4%
Died during all available follow-up time	62,998	10.3%	3,328	28.1%	26,229	14.2%
Died within 1 year after an infection	N/A	N/A	1,287	10.9%	3,687	2.0%

^1^Data are from the Danish National Health Survey

^2^Data are from the Danish Civil Registration System

^3^Data are from the Danish National Patient Registry

^4^Data are from the Danish National Prescription Registry

Selected characteristics of individuals with infections are presented in Table 1. Marked differences were observed between those with hospital-diagnosed or community-treated infections vs. all eligible individuals. For example, a larger proportion of those with community-treated infections were females, and those with hospital-diagnosed infections tended to be older compared to the other groups. Differences were observed in both self-reported and hospital-diagnosed underlying conditions (e.g., individuals with a hospital-diagnosed infection had higher prevalence of short- and long-term mental illness, hypertension, and cancer compared to those without an infection and those with community-treated infections). Prescription medication use was more common among individuals with rather than without infections of either type. Smoking status (19.8–21.7% were current smokers, 31.6–35.5% former smokers, 37.6–44.9% never smokers) and body mass index (1.9–3.1% were underweight, 40.2–43.2% normal weight, 31.1–33.7% overweight, and 15.8–18.1% obese), both of which are important variables associated with infections, generally had low missingness across all groups (3.7%–5.2% and 5.5%–7.5%, respectively). Mortality was highest among those with a hospital-diagnosed infection compared to those with a community-treated infection, and all eligible individuals.

Table 2 presents counts of the specific infections and types of anti-infectives in the cohort. Given that more than one infection was possible per eligible individual (i.e., counting all eligible infections), we identified 315,689 infections overall: 25,385 hospital-diagnosed and 290,304 community-treated. The most frequent hospital-diagnosed infection was respiratory infections (including pneumonia), which made up more than one-third of all diagnoses (34.9%; *n* = 8,858). Urinary tract infections were the second most frequent hospital-diagnosed infection (16.6%; *n* = 4,225). Most community-treated infections were linked to redeemed prescriptions for antibiotics (80.6%; *n* = 233,994). Examination of antibiotic type (data not shown) indicated that beta lactam antibiotics were redeemed for 68.0% of all infections identified according to antibiotic use; the next most frequent antibiotic was macrolides at 14.3%. The indication variable in the National Prescription Registry was complete for 61.0% of all anti-infectives used to define community-treated infections. The indication was 75.5% complete for antibiotics, but included both specific (e.g., otitis media) and unspecific (e.g., infection) indications. The variable was highly missing for most other anti-infective categories, and completeness across years varied substantially for antibiotics.

**Table 2:** Infection counts and type in The Danish Infection Cohort

	All survey years	
	No.	%
Total infections	315,689	100%
*Year of survey*		
2010	99,846	31.6%
2013	75,218	23.8%
2017	75,812	24.0%
2021	64,813	20.5%
*Hospital diagnosed infections*		
*Any diagnosis*	*25*,*385*	*100%*
Respiratory (including pneumonia)	8,858	34.9%
Urinary Tract	4,225	16.6%
Skin/Soft Tissue	1,971	7.8%
Eye and ear	1,411	5.6%
Sepsis	1,175	4.6%
Gastrointestinal	1,119	4.4%
Miscellaneous bacterial Infections	1,088	4.3%
Infectious complications of procedures, catheters etc.	889	3.5%
Intra-abdominal	739	2.9%
Obstetrical infections	707	2.8%
STIs	547	2.2%
Miscellaneous viral Infections	522	2.1%
Male genital infections	443	1.7%
Fungal	373	1.5%
Female pelvic infections	341	1.3%
Herpes viruses (zoster and simplex)	261	1.0%
Other infections or sequelae	171	0.7%
Septic arthritis, osteomyelitis, myositis	146	0.6%
CNS	146	0.6%
Heart Infections	126	0.5%
Parasitic Infections	91	0.4%
Tuberculosis	36	0.1%

Community treated infections^1,2^			Indication data complete
*Any anti-infective*	*290*,*304*	*100%*	*61.0%*
Antibiotics	233,994	80.6%	75.5%
Antiparasitics	19,812	6.8%	<1%
Antivirals^3^	20,014	6.9%	<1%
Antimycotics	16,426	5.7%	3.2%
Antimycobacterials	58	0.02%	8.6%

^1^Data are from the Danish National Prescription Registry

^2^Categories are based on ATC categorization

More granular information (i.e., by specific ATC) is available in the Prescription Registry

^3^Two types of antivirals were identified: neuraminidase inhibitors (J05AH*, *n*=136); nucleosides and nucleotides, excluding reverse transcriptase inhibitors (J05AB*, *n*=19,878)

## Discussion

Through the creation of the Danish Infection Cohort, we found that one-third of individuals in the four recent National Health Surveys had an infection within 1 year after survey response, yielding nearly 316,000 infections overall. The broad range of infection types included almost 9,000 hospital-diagnosed respiratory infections and more than 4,000 hospital-diagnosed urinary tract infections. The cohort received > 290,000 community prescriptions for anti-infectives during follow-up. We have presented selected aspects of the data for the cohort, both overall and by survey year. Depending on the specific study question, examining the cohort by age, sex, socioeconomic position, underlying medical condition, and other factors will be important.

The Danish National Health Survey provides a source of lifestyle data and other self-reported information not captured in the Danish national health registries. The survey has been used for a range of studies to date, and promises to enable many more studies as the data continue to accumulate. The survey data, combined with the comprehensive, longitudinal, and complete national registry data, uniquely enable studies on infectious disease epidemiology. The cohort we have created includes a wide range of infection types, a characteristic that is unique compared to other infection-based cohorts reported in the literature which are typically infection-specific (e.g., HIV). The nature of the cohort means it can be used to address a broad range of infectious disease questions. For example, while the relationship between weight and infection has been relatively well studied [3, 17–19], whether physical activity modifies this association is unclear. Also, recent research has shown that there are increased risks of post-acute and long-term cardiovascular outcomes following certain respiratory infections, but the studies to-date have lacked at least some information on key lifestyle and behavioral characteristics and/or did not take patient level socioeconomic position into account [20–22]. Further, and more broadly speaking, the role of socioeconomic position on infection risk and severity warrants continued and renewed examination [23, 24], and the new cohort is well positioned to serve this need.

Several studies have examined the Danish National Health Survey data with respect to the representativeness of the respondents, response rates, and agreement between self-reported information and national registry data. For the 2010–2021 surveys, the participation rate was 54%–60%, depending on the year, and the response rates were relatively low among some population groups, including young men, older women, unmarried individuals, and members of minority ethnic groups [10, 11, 25, 26]. Non-response can reduce generalizability and introduce bias, however, non-response weights can be applied to increase the generalizability of the survey respondents to the full Danish population [27–30]. That being said, internal validity is the priority for any epidemiologic study and is necessary for ensuring scientific generalizability [31]. In addition, imputing missing data for key study variables should be strongly considered [32]. The agreement between self-reported survey data on underlying conditions and information captured in the national registries has been found to vary according to the condition of interest [33, 34]. For some conditions, such as depression, the survey may be the better source of information than the registries, depending on the study question [19].

We broadly categorized infections into hospital-diagnosed and community-treated infections, by grouping diagnoses in infectious disease categories that we deemed most useful according to our experience and the high-level categories of anti-infectives in the ATC coding system. However, the identification of infections can and should be adjusted according to the specific study questions. For example, specific infections can be identified according to a combination of both hospital-diagnosed and community-treated infections. Some study questions might warrant identifying the actual medication dispensed, and/or leveraging the indication variable in the National Prescription Registry, when that information is complete and specific. The indication variable is incomplete for many medications, including prescriptions that were originally written as non-electronic (which was more common prior to October 2017 when electronic prescribing became mandatory) [12]. When that variable is complete, the validity is expected to be favorable. In an investigation by Harbi and Pottegård nearly all of the > 66,000 prescriptions with indications sampled for review were correctly coded with respect to the pharmacy source data, although non-specific indications were recorded for as many as one-third of some drugs and drug classes [12].

We used a 1-year post-survey timeframe to identify infections. We assume that responses to the survey questions on behavior and lifestyle factors (e.g., smoking, weight, physical activity) could reasonably be assumed to have been generally stable during that time. However, a longer post-survey assessment window might be appropriate for some studies. In addition, as the cohort is comprised of individuals identified over more than 15 years, it is possible that changes in medical care and secular trends will be relevant to certain study questions. Examining the impact of calendar time may therefore be important.

We identified infections according to the occurrence of a single diagnosis code in the National Patient Registry or one dispensing from a community pharmacy. Because the National Patient Registry contains only hospital-based care, the diagnosed infections in this cohort were likely relatively severe. In addition, as occurs in many so-called real-world data sources, misclassification is expected. Given the hospital setting of the National Patient Registry, positive predictive values (PPV) have generally been high for coded diagnoses that have been studied, although the sensitivity, when assessed for specific infections, has been low. For example, diagnoses for serious infections among patients with cancer have been identified by primary diagnosis codes in the Danish Patient Registry in one study [35]. The PPV for any infection, regardless of type, was found to be 98% (95% CI: 96%–99%), according to 266 chart reviews, although when the specific type of infection was considered, the overall PPV was 80% (95% CI: 75%–85%) and varied by infection type. In a study of early-pandemic COVID-19 diagnosis codes, the PPV was 99% [36], but the PPV is likely to have changed over time. One group identified adults hospitalized with a positive test for respiratory syncytial virus in 2015–2018 and reported a PPV for respiratory syncytial virus diagnosis codes of 95.5% (95% CI: 93.1%–97.2%) in 440 patients, but the sensitivity was low, at 42.4% (95% CI: 39.3%–45.6%) [37]. For influenza diagnosis codes in the National Patient Registry, Hønge and colleagues have estimated an overall PPV of 87.9% (95% CI: 87.4%–88.3%) and have observed the highest PPV among older patients and those diagnosed in more recent years (95.8% in 2022 vs. 52.4% in 2012) [38]. However, low sensitivity was again reported: of > 33,000 hospitalizations with a positive influenza test, 48.5% had an influenza code in the registry. Importantly, given changing testing practices for respiratory viruses in Danish hospitals (e.g., routine testing of some patients regardless of symptom status), particularly since the COVID-19 pandemic, if laboratory-confirmed infections are included in a future study, asymptomatic patients might potentially be identified; therefore, care is necessary in defining infections for different study aims. Further, Gradel and colleagues have found that one-third of patients with bacteremia in 2000–2011 had a corresponding diagnosis code in the Danish National Patient Registry [39].

Other limitations to consider include potential misclassification of infection status – as well as some baseline covariates – as we assumed a survey response date of May 1 for all respondents. The specific timing of information from the survey may therefore be misclassified with respect to infection (e.g., someone who responded to the survey on August 1 and not May 1 of their survey year and who had an infection in July would have reported their survey information after their infection). In addition, infections could be incident or prevalent in this cohort. Studies that leverage this cohort may adjust this approach depending on the study question. Future studies might also consider including multiple surveys from those who participated in more than one (9% of respondents to date).

Linkage of data from this cohort to data from *additional* Danish registries should be considered according to study needs. The registries available in Denmark have been outlined by others [40, 41]. The registries potentially most relevant to infectious disease studies include the Danish Register of Causes of Death [42], the Danish National Hospital Medication Register [43], and various regional and national laboratory test result sources, including the national Register of Laboratory Results for Research [24–26]. Household level income, employment status, and other socioeconomic and demographic data are available from Statistics Denmark [47, 48].

## Conclusions

The Danish Infection Cohort—containing nearly 200,000 individuals with medically treated infections in the 1-year post-survey to date, more than 315,000 infections overall, and a broad range of infection types—should provide a valuable resource for future studies. We plan to update the cohort as the data from those included in the 2023 and 2025 National Health Survey become available. Because of the combination of self-reported lifestyle, behavioral, and health characteristics from the surveys, and the longitudinality and depth of the various Danish registries, this cohort can provide unique insights into important questions regarding the epidemiology, risk factors, natural history, prognosis, and sequelae of various infectious diseases.

## Supplementary Information

Below is the link to the electronic supplementary material.

Supplementary Material 1

## Data Availability

The National Health Surveys in 2010–2021 were funded by the Capital Region, Region Zealand, Region Southern Denmark, Region Central Denmark, Region North Denmark, Ministry of the Interior and Health, and the National Institute of Public Health, University of Southern Denmark. Data were collected by the five regions and the National Institute of Public Health, University of Southern Denmark.
