# Supplementary material for: The Danish Infection Cohort: a resource for population based infectious disease epidemiology
Source: Eur J Epidemiol. 2026 May 27;41(5):681–91. doi: 10.1007/s10654-026-01410-5 (PMC13332955; doi:10.1007/s10654-026-01410-5)
Supplement: Supplementary file 1 — Supplementary Material 1 [file 10654_2026_1410_MOESM1_ESM.docx]

**Supplemental Table 1. Danish National Health Survey participation by sex and age, 2010-2021**

|  | **2010** | | **2013** | | **2017** | | **2021** | |
| --- | --- | --- | --- | --- | --- | --- | --- | --- |
| **Total invited** | 298,550 | | 300,450 | | 311,157 | | 324,000 | |
| **Total respondents** | 177,639 (59.5%) | | 162,283 (54.0%) | | 183,372 (58.7%) | | 183,708 (56.7%) | |
|  | | | | | | | | |
| **Men** | No. invited | Responded (%) | No. invited | Responded (%) | No. invited | Responded (%) | No. invited | Responded (%) |
| Total | 146,791 | 55.5 | 147,908 | 50.4 | 153,276 | 54.8 | 160,019 | 52.0 |
| 16-24 years | 19,594 | 42.4 | 21,506 | 34.3 | 21,600 | 39.0 | 21,521 | 37.0 |
| 25-34 years | 17,560 | 44.9 | 17,437 | 36.3 | 21,514 | 38.4 | 23,973 | 32.0 |
| 35-44 years | 25,474 | 51.1 | 22,627 | 44.3 | 24,037 | 46.3 | 22,398 | 41.0 |
| 45-54 years | 26,898 | 56.3 | 27,303 | 50.8 | 27,464 | 55.9 | 27,398 | 51.6 |
| 55-64 years | 26,964 | 63.5 | 24,878 | 59.8 | 24,051 | 66.1 | 25,743 | 62.1 |
| 65-74 years | 18,704 | 69.9 | 21,712 | 67.1 | 22,165 | 74.2 | 22,004 | 74.0 |
| ≥75 years | 11,597 | 60.1 | 12,445 | 60.3 | 12,445 | 60.3 | 16,982 | 70.9 |
|  | | | | | | | | |
| **Women** | No. invited | Responded (%) | No. invited | Responded (%) | No. invited | Responded (%) | No. invited | Responded (%) |
| Total | 151,759 | 63.4 | 152,542 | 57.5 | 157,881 | 62.6 | 163,981 | 61.2 |
| 16-24 years | 17,995 | 57.0 | 20,017 | 48.1 | 20,204 | 51.9 | 19,939 | 52.0 |
| 25-34 years | 17,557 | 59.2 | 16,785 | 49.3 | 20,616 | 52.2 | 22,988 | 46.3 |
| 35-44 years | 25,529 | 64.6 | 22,888 | 55.4 | 23,474 | 59.6 | 22,273 | 54.2 |
| 45-54 years | 26,635 | 67.2 | 27,016 | 61.1 | 27,438 | 66.8 | 26,977 | 63.1 |
| 55-64 years | 26,246 | 70.3 | 25,042 | 65.6 | 24,404 | 72.4 | 26,254 | 71.3 |
| 65-74 years | 20,023 | 69.3 | 22,809 | 66.4 | 23,325 | 73.7 | 23,393 | 76.1 |
| ≥75 years | 17,774 | 49.4 | 17,985 | 50.3 | 18,420 | 56.2 | 22,157 | 62.2 |

Data sources for Supplemental Table 1

- Christensen AI, Lau CJ, Kristensen PL, Johnsen SB, Wingstrand A, Friis K, Davidsen M, Andreasen AH. The Danish National Health Survey: Study design, response rate and respondent characteristics in 2010, 2013 and 2017. Scand J Public Health. 2022 Mar;50(2):180-188.
- Christensen AI, Lau CJ, Kristensen PL, Poulsen HS, Breinholt Larsen F. 35 Years of health surveys in Denmark: a backbone of public health practice and research. Scand J Public Health. 2022 Nov;50(7):914-918. doi: 10.1177/14034948221083113. Epub 2022 May 12. PMID: 35548941.
- <https://www.danskernessundhed.dk/Baggrund.html>

**Supplemental Table 2. International Classification of Diseases, 10^th^ Revision, Clinical Modification (ICD-10-CM), codes used to identify hospital-diagnosed infections**

| **Infection category^1^** | **ICD-10 code^2^** | **Description** | |
| --- | --- | --- | --- |
| Respiratory | J00* | Acute nasopharyngitis | |
| Respiratory | J01* | Acute sinusitis | |
| Respiratory | J02* | Acute pharyngitis | |
| Respiratory | J03* | Acute tonsillitis | |
| Respiratory | J04* | Acute laryngitis and tracheitis | |
| Respiratory | J05* | Acute obstructive laryngitis [croup] and epiglottitis | |
| Respiratory | J06* | Acute upper respiratory infections of multiple and unspecified sites | |
| Respiratory | J36.9 | Peritonsillar abscess | |
| Respiratory | J39.0 | Retropharyngeal and parapharyngeal abscess | |
| Respiratory | J39.1 | Other abscess of pharynx | |
| Respiratory | J09* | Influenza caused by identified zoonotic or pandemic types of influenza virus | |
| Respiratory | J10.1 | Influenza with other respiratory manifestations caused by other type of influenza virus | |
| Respiratory | J10.1A | Influenza with laryngitis caused by other influenza virus type | |
| Respiratory | J10.1B | Influenza with pharyngitis caused by other type of influenza virus | |
| Respiratory | J10.1C | Influenza with pleural exudate caused by other influenza virus type | |
| Respiratory | J10.8 | Influenza with other manifestation caused by other type of influenza virus | |
| Respiratory | J10.8A | Influenza with gastroenteritis caused by other influenza virus type | |
| Respiratory | J11.1 | Influenza with other respiratory manifestations without influenza virus detected | |
| Respiratory | J11.1A | Influenza with laryngitis without influenza virus detected | |
| Respiratory | J11.1B | Influenza with pharyngitis without influenza virus detected | |
| Respiratory | J11.1C | Influenza with pleural exudate without influenza virus detected | |
| Respiratory | J11.8 | Influenza with other manifestation without detected influenza virus | |
| Respiratory | J11.8B | Influenza with gastroenteritis without influenza virus detected | |
| Respiratory | J11.8D | Otitis media due to influenza UNS | |
| Respiratory | J20* | Acute bronchitis | |
| Respiratory | J21* | Acute bronchiolitis | |
| Respiratory | J22* | Unspecified acute lower respiratory infection | |
| Respiratory | J44.0 | Chronic obstructive pulmon disease with (acute) lower resp infct | |
| Respiratory | J86* | Pyothorax | |
| Respiratory | J38.3C | Cellulitis of vocal cords | |
| Respiratory | J38.3D | Abscess of vocal cords | |
| Respiratory | J38.7B | Perichondritis of larynx | |
| Respiratory | J38.7F | Cellulitis of larynx | |
| Respiratory | J38.7G | Abscess of larynx | |
| Respiratory | B97.4 | Respiratory syncytial virus as the cause of diseases classified elsewhere | |
| Respiratory | B97.0 | Adenovirus as a cause of disease | |
| Respiratory | B97.1 | Enterovirus as a cause of disease | |
| Respiratory | B97.2 | Coronavirus as a cause of disease | |
| Respiratory | B34.0 | Adenovirus infection without site specified | |
| Respiratory | B34.1 | Enterovirus infection without site specified | |
| Respiratory | B34.2 | Coronavirus infection without site specified | |
| Respiratory | B34.2A | COVID-19 infection without indication of location | |
| Respiratory | B97.2A | COVID-19 severe acute respiratory syndrome | |
| Respiratory | B97.2B | Multisystem inflammatory syndrome associated with COVID-19 | |
| Respiratory | B97.2B1 | Multisystem inflammatory syndrome in children | |
| Respiratory | U07.1 | Emergency use of U07.1 \| COVID-19, virus identified | |
| Respiratory | U10 | Multisystem inflammatory syndrome associated with COVID-19 | |
| Respiratory | U10.9 | Multisystem inflammatory syndrome associated with COVID-19, unspecified | |
| Gastrointestinal | A00* | Cholera | |
| Gastrointestinal | A01* | Typhoid and paratyphoid fevers | |
| Gastrointestinal | A02* (except A02.1) | Other salmonella infections | |
| Gastrointestinal | A03* | Shigellosis | |
| Gastrointestinal | A04* | Other bacterial intestinal infections | |
| Gastrointestinal | A05* | Other bacterial foodborne intoxications, not elsewhere classified | |
| Gastrointestinal | A06* | Amebiasis | |
| Gastrointestinal | A07* | Other protozoal intestinal diseases | |
| Gastrointestinal | A08* | Viral and other specified intestinal infections | |
| Gastrointestinal | A09* | Infectious gastroenteritis and colitis, unspecified | |
| Urinary Tract | N10* | Acute pyelonephritis | |
| Urinary Tract | N12* | Tubulo-interstitial nephritis, not spcf as acute or chronic | |
| Urinary Tract | N15.1 | Renal and perinephric abscess | |
| Urinary Tract | N15.9 | Renal tubulo-interstitial disease, unspecified | |
| Urinary Tract | N30.0 | Acute cystitis | |
| Urinary Tract | N30.3 | Trigonitis | |
| Urinary Tract | N30.8 | Other cystitis | |
| Urinary Tract | N30.9 | Cystitis, unspecified | |
| Urinary Tract | N33.0 | Bladder disorders in diseases classified elsewhere- Tuberculous cystitis | |
| Urinary Tract | N34* | Urethritis and urethral syndrome | |
| Urinary Tract | N39.0 | Urinary tract infection, site not specified | |
| Urinary Tract | N08.0 | Glomerulonephropathy in infectious or parasitic disease classified elsewhere | |
| Urinary Tract | N13.6 | Pyonephrosis | |
| Urinary Tract | N16.0 | Nephropathy in infectious or parasitic disease classified elsewhere | |
| Urinary Tract | N28.8D | Cystic pyelitis | |
| Urinary Tract | N28.8E | Pyeloureteritis cystica | |
| Urinary Tract | N28.8F | Cystic ureteritis | |
| Urinary Tract | N29.0 | Syphilitic kidney disease UNS | |
| Urinary Tract | N29.1 | Disease of kidney or ureter in infectious or parasitic disease classified elsewhere | |
| Skin/Soft Tissue | A46* | Erysipelas | |
| Skin/Soft Tissue | K12.2 | Cellulitis and abscess of mouth | |
| Skin/Soft Tissue | K13.0A | Abscess of the labia majora | |
| Skin/Soft Tissue | K61* | Abscess of anal and rectal regions | |
| Skin/Soft Tissue | M72.6 | Necrotizing fasciitis | |
| Skin/Soft Tissue | L01* | Impetigo | |
| Skin/Soft Tissue | L08* | Other local infections of skin and subcutaneous tissue | |
| Sepsis | A40* | Streptococcal sepsis | |
| Sepsis | A41* | Other sepsis | |
| Sepsis | B37.7 | Candidal sepsis | |
| Sepsis | A32.7 | Listerial sepsis | |
| Sepsis | A54.8G | Gonorrheal sepsis | |
| Sepsis | A02.1 | Salmonella sepsis | |
| Sepsis | A22.7 | Anthrax sepsis | |
| Sepsis | A26.7 | Erysipelothrix sepsis | |
| Sepsis | A42.7 | Actinomycotic sepsis | |
| Sepsis | A28.2B | Yersinia sepsis | |
| Sepsis | A49.9A | Bacteremia UNS | |
| Sepsis | A39.4 | Meningococcal bacteremia UNS | |
| Pneumonia | J12* | Viral pneumonia, not elsewhere classified | |
| Pneumonia | J13* | Pneumonia due to Streptococcus pneumoniae | |
| Pneumonia | J14* | Pneumonia due to Hemophilus influenzae | |
| Pneumonia | J15* | Bacterial pneumonia, not elsewhere classified | |
| Pneumonia | J16* | Pneumonia due to other infectious organisms, not elsewhere classified | |
| Pneumonia | J17* | Pneumonia in diseases classified elsewhere | |
| Pneumonia | J18* | Pneumonia, unspecified organism | |
| Pneumonia | J85.1 | Abscess of lung with pneumonia | |
| Pneumonia | J10.0 | Influenza with pneumonia caused by other type of influenza virus | |
| Pneumonia | J11.0 | Influenza with pneumonia without influenza virus detected | |
| CNS | G00* | Bacterial meningitis, not elsewhere classified | |
| CNS | G01* | Meningitis in bacterial diseases classified elsewhere | |
| CNS | G02* | Meningitis in oth infec/parastc diseases classd elswhr | |
| CNS | G03* | Meningitis due to other and unspecified causes | |
| CNS | G04* | Encephalitis, myelitis and encephalomyelitis | |
| CNS | G05* | Encephalitis, myelitis and encephalomyelitis in diseases classified elsewhere | |
| CNS | G06* | Intracranial and intraspinal abscess and granuloma | |
| CNS | G07* (except G07.9J, G07.9K) | Intcrn & intraspinal abscs & granuloma in dis classd elswhr | |
| CNS | A80* | Acute poliomyelitis | |
| CNS | A81* | Atypical virus infections of central nervous system | |
| CNS | A82* | Rabies | |
| CNS | A83* | Mosquito-borne viral encephalitis | |
| CNS | A84* | Tick-borne viral encephalitis | |
| CNS | A85* | Other viral encephalitis, not elsewhere classified | |
| CNS | A86* | Unspecified viral encephalitis | |
| CNS | A87* | Viral meningitis | |
| CNS | A88* | Other viral infections of central nervous system, not elsewhere classified | |
| CNS | A89* | Unspecified viral infection of central nervous system | |
| CNS | A39* (except A39.4) | Meningococcal infection | |
| Heart Infections | I00* | Rheumatic fever without heart involvement | |
| Heart Infections | I01* | Rheumatic fever with heart involvement | |
| Heart Infections | I02* | Rheumatic chorea | |
| Heart Infections | I30.1 | Infective pericarditis | |
| Heart Infections | I32.0 | Pericarditis in diseases classified elsewhere | |
| Heart Infections | I33* | Acute and subacute endocarditis | |
| Heart Infections | I38* | Endocarditis, valve unspecified | |
| Heart Infections | I40.0 | Infective myocarditis | |
| Heart Infections | I39.8 | Endocarditis and heart valve disord in dis classd elswhr | |
| Heart Infections | B37.6 | Candidal endocarditis | |
| Eye and Ear | H00* | Hordeolum and chalazion | |
| Eye and Ear | H01.0* | Blepharitis | |
| Eye and Ear | H03.0 | Affection of eyelid due to parasitic infestation classified elsewhere | |
| Eye and Ear | H03.1 | Affection of eyelid in other infectious disease classified elsewhere | |
| Eye and Ear | H04.3* | Acute and unspecified inflammation of lacrimal passages | |
| Eye and Ear | H05.0* | Acute inflammation of orbit | |
| Eye and Ear | H06.1 | Parasitic infection of eye socket in disease classified elsewhere | |
| Eye and Ear | H10* | Conjunctivitis | |
| Eye and Ear | H13.0 | Filariasis of the conjunctiva | |
| Eye and Ear | H13.1 | Conjunctivitis in infectious or parasitic disease classified elsewhere | |
| Eye and Ear | H15.0* | Scleritis | |
| Eye and Ear | H19.2 | Keratitis and keratoconjunctivitis in other infectious or parasitic diseases classified elsewhere | |
| Eye and Ear | H22.0 | Iridocyclitis in infectious or parasitic disease classified elsewhere | |
| Eye and Ear | H32.0 | Chorioretinitis in infectious or parasitic disease classified elsewhere | |
| Eye and Ear | H44.0* | Purulent endophthalmitis | |
| Eye and Ear | H44.1* | Other endophthalmitis | |
| Eye and Ear | H60.0* | Abscess of external ear | |
| Eye and Ear | H60.1* | Cellulitis of external ear | |
| Eye and Ear | H60.2* | Malignant otitis externa | |
| Eye and Ear | H60.3* | Other infective otitis externa | |
| Eye and Ear | H60.9* | Unspecified otitis externa | |
| Eye and Ear | H61.0* | Chondritis and perichondritis of external ear | |
| Eye and Ear | H62.0 | External otitis in bacterial disease classified elsewhere | |
| Eye and Ear | H62.1 | External otitis in viral disease classified elsewhere | |
| Eye and Ear | H62.2 | External otitis due to mycosis classified elsewhere | |
| Eye and Ear | H62.3 | Otitis externa in other infectious or parasitic disease classified elsewhere | |
| Eye and Ear | H65.0* | Acute serous otitis media | |
| Eye and Ear | H65.1* | Other acute nonsuppurative otitis media | |
| Eye and Ear | H65.9* | Otitis media without suppuration UNS | |
| Eye and Ear | H66.0* | Acute suppurative otitis media | |
| Eye and Ear | H66.4* | Suppurative otitis media, unspecified | |
| Eye and Ear | H66.9* | Otitis media, unspecified | |
| Eye and Ear | H67.0 | Otitis media in diseases classified elsewhere | |
| Eye and Ear | H67.1 | Otitis media in viral disease classified elsewhere | |
| Eye and Ear | H67.8 | Otitis media in other disease classified elsewhere | |
| Eye and Ear | H68.0 | Otosalpingitis (68.0) | |
| Eye and Ear | H70.0* | Acute mastoiditis | |
| Eye and Ear | H70.2* | Petrositis | |
| Eye and Ear | H70.9* | Unspecified mastoiditis | |
| Eye and Ear | H73.0* | Acute tympanitis | |
| Eye and Ear | H75.0* | Mastoiditis in infectious and parasitic diseases classified elsewhere | |
| Eye and Ear | H83.0* | Labyrinthitis | |
| Eye and Ear | H94.0* | Acoustic neuritis in infectious and parasitic diseases classified elsewhere | |
| Fungal | B35* | Dermatophytosis | |
| Fungal | B36* | Other superficial mycoses | |
| Fungal | B37* (except B37.6, B37.7) | Candidiasis | |
| Fungal | B38* | Coccidioidomycosis | |
| Fungal | B39* | Histoplasmosis | |
| Fungal | B40* | Blastomycosis | |
| Fungal | B41* | Paracoccidioidomycosis | |
| Fungal | B42* | Sporotrichosis | |
| Fungal | B43* | Chromomycosis and pheomycotic abscess | |
| Fungal | B44* | Aspergillosis | |
| Fungal | B45* | Cryptococcosis | |
| Fungal | B46* | Zygomycosis | |
| Fungal | B47* | Mycetoma | |
| Fungal | B48* | Other mycoses, not elsewhere classified | |
| Fungal | B49* | Unspecified mycosis | |
| Tuberculosis | A15* | Respiratory tuberculosis | |
| Tuberculosis | A17* | Tuberculosis of nervous system | |
| Tuberculosis | A18* | Tuberculosis of other organs | |
| Tuberculosis | A19* | Miliary tuberculosis | |
| HIV | B20* | Human immunodeficiency virus [HIV] disease | |
| HIV | B22* | Human immunodeficiency virus (HIV) disease with other specified diseases | |
| HIV | B23* | Other conditions of HIV disease | |
| HIV | B24* | HIV disease and AIDS without further specification | |
| Viral hepatitis | B15* | Acute hep A | |
| Viral hepatitis | B16* | Acute hep B | |
| Viral hepatitis | B17* | Other acute viral hepatitis | |
| Viral hepatitis | B18* | Chronic viral hepatitis | |
| Viral hepatitis | B19* | Viral hepatitis, unspecified | |
| Sexually transmitted infections | A50* | Congenital syphilis | |
| Sexually transmitted infections | A51* | Early syphilis | |
| Sexually transmitted infections | A52* | Late syphilis | |
| Sexually transmitted infections | A53* | Other and unspecified syphilis | |
| Sexually transmitted infections | A54* (except A54.8G) | Gonococcal infection | |
| Sexually transmitted infections | A55* | Chlamydial lymphogranuloma (venereum) | |
| Sexually transmitted infections | A56* | Other sexually transmitted chlamydial diseases | |
| Sexually transmitted infections | A57* | Chancroid | |
| Sexually transmitted infections | A58* | Granuloma inguinale | |
| Sexually transmitted infections | A59* | Trichomoniasis | |
| Sexually transmitted infections | A60* | Anogenital herpesviral [herpes simplex] infections | |
| Sexually transmitted infections | A63* | Other predominantly sexually transmitted diseases, not elsewhere classified | |
| Sexually transmitted infections | A64* | Unspecified sexually transmitted disease | |
| Miscellaneous bacterial Infections | A20* | Plague | |
| Miscellaneous bacterial Infections | A21* | Tularemia | |
| Miscellaneous bacterial Infections | A22* (except A22.7) | Anthrax | |
| Miscellaneous bacterial Infections | A23* | Brucellosis | |
| Miscellaneous bacterial Infections | A24* | Glanders and melioidosis | |
| Miscellaneous bacterial Infections | A25* | Rat-bite fevers | |
| Miscellaneous bacterial Infections | A26* (except A26.7) | Erysipeloid | |
| Miscellaneous bacterial Infections | A27* | Leptospirosis | |
| Miscellaneous bacterial Infections | A28* (except A28.2B) | Other zoonotic bacterial diseases, not elsewhere classified | |
| Miscellaneous bacterial Infections | A30* | Leprosy [Hansen's disease] | |
| Miscellaneous bacterial Infections | A31* | Infection due to other mycobacteria | |
| Miscellaneous bacterial Infections | A32* (except A32.7) | Listeriosis | |
| Miscellaneous bacterial Infections | A33* | Tetanus neonatorum | |
| Miscellaneous bacterial Infections | A34* | Obstetrical tetanus | |
| Miscellaneous bacterial Infections | A35* | Other tetanus | |
| Miscellaneous bacterial Infections | A36* | Diphtheria | |
| Miscellaneous bacterial Infections | A37* | Whooping cough | |
| Miscellaneous bacterial Infections | A38* | Scarlet fever | |
| Miscellaneous bacterial Infections | A42.0 | Pulmonary actinomycosis | |
| Miscellaneous bacterial Infections | A42.1 | Abdominal actinomycosis | |
| Miscellaneous bacterial Infections | A42.2 | Cervicofacial actinomycosis | |
| Miscellaneous bacterial Infections | A42.8* | Other forms of actinomycosis | |
| Miscellaneous bacterial Infections | A42.9 | Actinomycosis, unspecified | |
| Miscellaneous bacterial Infections | A43* | Nocardiosis | |
| Miscellaneous bacterial Infections | A44* | Bartonellosis | |
| Miscellaneous bacterial Infections | A48* | Other bacterial diseases, not elsewhere classified | |
| Miscellaneous bacterial Infections | A49* (except A49.9A) | Bacterial infection of unspecified site | |
| Miscellaneous bacterial Infections | A65* | Nonvenereal syphilis | |
| Miscellaneous bacterial Infections | A66* | Yaws | |
| Miscellaneous bacterial Infections | A67* | Pinta [carate] | |
| Miscellaneous bacterial Infections | A68* | Relapsing fevers | |
| Miscellaneous bacterial Infections | A69* | Other spirochetal infections | |
| Miscellaneous bacterial Infections | A70* | Chlamydia psittaci infections | |
| Miscellaneous bacterial Infections | A71* | Trachoma | |
| Miscellaneous bacterial Infections | A74* | Other diseases caused by chlamydiae | |
| Miscellaneous bacterial Infections | A75* | Typhus fever | |
| Miscellaneous bacterial Infections | A77* | Spotted fever [tick-borne rickettsioses] | |
| Miscellaneous bacterial Infections | A78* | Q fever | |
| Miscellaneous bacterial Infections | A79* | Other rickettsioses | |
| Miscellaneous bacterial Infections | B95* | Streptococci and staphylococci as causes of disease | |
| Miscellaneous bacterial Infections | B96* | Other bacteria as a cause of disease | |
| Miscellaneous viral infections | A92* | Other mosquito-borne viral fevers | |
| Miscellaneous viral infections | A93* | Other arthropod-borne viral fevers, not elsewhere classified | |
| Miscellaneous viral infections | A94* | Unspecified arthropod-borne viral fever | |
| Miscellaneous viral infections | A95* | Yellow fever | |
| Miscellaneous viral infections | A96* | Arenaviral hemorrhagic fever | |
| Miscellaneous viral infections | A97* | Dengue fever | |
| Miscellaneous viral infections | A98* | Other viral hemorrhagic fevers, not elsewhere classified | |
| Miscellaneous viral infections | A99* | Unspecified viral hemorrhagic fever | |
| Miscellaneous viral infections | B03* | Smallpox | |
| Miscellaneous viral infections | B04* | Monkeypox | |
| Miscellaneous viral infections | B05* | Measles | |
| Miscellaneous viral infections | B06* | Rubella | |
| Miscellaneous viral infections | B07* | Viral warts | |
| Miscellaneous viral infections | B08* | Other viral infections characterized by skin and mucous membrane lesions, not elsewhere classified | |
| Miscellaneous viral infections | B09* | Unsp viral infection with skin and mucous membrane lesions | |
| Miscellaneous viral infections | B25* | Cytomegaloviral disease | |
| Miscellaneous viral infections | B26* | Mumps | |
| Miscellaneous viral infections | B27* | Infectious mononucleosis | |
| Miscellaneous viral infections | B30* | Viral conjunctivitis | |
| Miscellaneous viral infections | B33* | Other viral diseases, not elsewhere classified | |
| Miscellaneous viral infections | B34* (except B34.0, B34.1, B34.2, B34.2A) | Viral infection of unspecified site | |
| Miscellaneous viral infections | B97.3 | Retrovirus as a cause of disease | |
| Miscellaneous viral infections | B97.5 | Reovirus as a cause of disease | |
| Miscellaneous viral infections | B97.6 | Parvovirus as a cause of disease | |
| Miscellaneous viral infections | B97.7 | Papillomavirus as a cause of disease | |
| Miscellaneous viral infections | B97.8 | Other virus as a cause of disease | |
| Herpes viruses (zoster and simplex) | B00* | Herpes simplex infection | |
| Herpes viruses (zoster and simplex) | B01* | Chickenpox | |
| Herpes viruses (zoster and simplex) | B02* | Shingles | |
| Parasitic Infections | B50* | Plasmodium falciparum malaria | |
| Parasitic Infections | B51* | Plasmodium vivax malaria | |
| Parasitic Infections | B52* | Plasmodium malariae-malaria | |
| Parasitic Infections | B53* | Plasmodium ovale malaria | |
| Parasitic Infections | B54* | Malaria without further specification | |
| Parasitic Infections | B55* | Leishmaniasis | |
| Parasitic Infections | B56* | African sleeping sickness | |
| Parasitic Infections | B57* | Chagas' disease | |
| Parasitic Infections | B58* | Toxoplasmosis | |
| Parasitic Infections | B60* | Other diseases caused by protozoa IKA | |
| Parasitic Infections | B64* | Diseases caused by protozoa UNS | |
| Parasitic Infections | B65* | Schistosomiasis | |
| Parasitic Infections | B66* | Other icteric infections | |
| Parasitic Infections | B67* | Echinococcosis | |
| Parasitic Infections | B68* | Tapeworm infection | |
| Parasitic Infections | B69* | Cysticercosis | |
| Parasitic Infections | B70* | Diphyllobothriasis and sparganosis | |
| Parasitic Infections | B71* | Dipylidiasis | |
| Parasitic Infections | B72* | Dracunculiasis | |
| Parasitic Infections | B73* | Onchocerciasis | |
| Parasitic Infections | B74* | Filariasis | |
| Parasitic Infections | B75* | Trichinosis | |
| Parasitic Infections | B76* | Hookworm infestation | |
| Parasitic Infections | B77* | Ascariasis | |
| Parasitic Infections | B78* | Strongyloidiasis | |
| Parasitic Infections | B79* | Whipworm infection | |
| Parasitic Infections | B80* | Infection with pinworms | |
| Parasitic Infections | B81* | Other intestinal worm infestations IKA | |
| Parasitic Infections | B82* | Intestinal infestations with parasites, unspecified | |
| Parasitic Infections | B83* | Other worm diseases | |
| Parasitic Infections | B85* | Infestation with lice | |
| Parasitic Infections | B87* | Myiasis | |
| Parasitic Infections | B88* | Other arthropod infestations | |
| Parasitic Infections | B89* | Disease caused by parasite UNS | |
| Intra-abdominal | K65.0* | Acute peritonitis | |
| Intra-abdominal | K65.9 | Peritonitis NOS | |
| Intra-abdominal | K67* | Disorders of the peritoneum in infectious diseases classified elsewhere | |
| Intra-abdominal | K75.1 | Pylephlebitis | |
| Intra-abdominal | K80.3* | Stones in bile duct with cholangitis | |
| Intra-abdominal | K80.4* | Stones in bile duct with cholecystitis | |
| Intra-abdominal | K81.9 | Cholecystitis UNS | |
| Intra-abdominal | K83.0* | Cholangitis | |
| Intra-abdominal | K85.9 | Acute pancreatitis UNS | |
| Male genital infections | N41* | Inflammation of the prostate gland | |
| Male genital infections | N45* | Inflammation of testicle and epididymis | |
| Male genital infections | N48.1* (except N48.1D) | Balanoposthitis | |
| Male genital infections | N48.2* | Other inflammatory condition of penis | |
| Male genital infections | N49* | Other inflammatory conditions of the male genital organs IKA | |
| Male genital infections | N51.1K | Epididymitis in diseases classified elsewhere | |
| Male genital infections | N51.1L | Orchitis in diseases classified elsewhere | |
| Male genital infections | N51.2 | Balanitis in diseases classified elsewhere | |
| Female pelvic infections | N70.0* | Acute salpingitis or oophoritis | |
| Female pelvic infections | N70.9* | Salpingitis or oophoritis UNS | |
| Female pelvic infections | N71.0* | Acute endometritis | |
| Female pelvic infections | N71.9 | Endometritis UNS | |
| Female pelvic infections | N72* (except N72.9B, N72.9E) | Inflammatory conditions of the cervix | |
| Female pelvic infections | N73.0* | Acute parametritis or phlegmon of female pelvis | |
| Female pelvic infections | N73.2* | Parametritis or phlegmon of female pelvis UNS | |
| Female pelvic infections | N73.3* | Acute female pelvic peritonitis | |
| Female pelvic infections | N73.5* | Female pelvic peritonitis NOS | |
| Female pelvic infections | N73.8* (except N73.8B) | Other infection of female pelvis | |
| Female pelvic infections | N73.9 | Female pelvic infection UNS | |
| Female pelvic infections | N74* | Pelvic inflammatory disease in diseases classified elsewhere | |
| Female pelvic infections | N75.1 | Abscess of Bartholin's gland | |
| Female pelvic infections | N75.8A | Acute bartholinitis | |
| Female pelvic infections | N75.8C | Bartholinitis UNS | |
| Female pelvic infections | N76.0 | Acute vaginitis | |
| Female pelvic infections | N76.2* | Acute vulvitis | |
| Female pelvic infections | N76.4 | Abscess of the vulva | |
| Female pelvic infections | N76.8* | Other inflammatory disease of vagina or vulva | |
| Female pelvic infections | N77* (except N77.8*) | Ulcers and inflammation of vagina and external female genitalia in diseases classified elsewhere | |
| Obstetrical infections | O23* | Pelvic and urinary tract infections in pregnancy | |
| Obstetrical infections | O26.4 | Herpes gestationis | |
| Obstetrical infections | O41.1* | Infection of amniotic cavity and membranes | |
| Obstetrical infections | O74.0 | Aspiration pneumonia during anesthesia at birth | |
| Obstetrical infections | O75.3* | Other infection during childbirth | |
| Obstetrical infections | O85* | Puerperal fever | |
| Obstetrical infections | O86* | Other infections during the puerperium | |
| Obstetrical infections | O88.3 | Obstetric septic embolism | |
| Obstetrical infections | O91* | Infections of mammary gland and nipple associated with childbirth | |
| Obstetrical infections | O98* | Maternal infections and parasitic diseases classified to other chapters but complicating pregnancy, childbirth and the puerperium | |
| Septic arthritis, osteomyelitis, myositis | M00* | Purulent arthritis | |
| Septic arthritis, osteomyelitis, myositis | M01* | Arthritis in infectious and parasitic diseases classified elsewhere | |
| Septic arthritis, osteomyelitis, myositis | M86.0 | Acute hematogenous osteomyelitis | |
| Septic arthritis, osteomyelitis, myositis | M86.1 | Other acute osteomyelitis | |
| Septic arthritis, osteomyelitis, myositis | M86.8* | Other osteomyelitis | |
| Septic arthritis, osteomyelitis, myositis | M86.9* | Osteomyelitis UNS | |
| Septic arthritis, osteomyelitis, myositis | M63.0 | Myositis in bacterial disease classified elsewhere | |
| Septic arthritis, osteomyelitis, myositis | M63.2 | Myositis in other infectious diseases classified elsewhere | |
| Infectious complications of procedures, catheters etc. | T80.2* | Infection following infusion, transfusion or injection UNS | |
| Infectious complications of procedures, catheters etc. | T81.4* | Post-procedural infection IKA | |
| Infectious complications of procedures, catheters etc. | T82.6* | Infection or inflammation around the prosthetic heart valve | |
| Infectious complications of procedures, catheters etc. | T82.7* | Infection or inflammation around other implant or graft in the heart or blood vessels | |
| Infectious complications of procedures, catheters etc. | T83.5* | Infection or inflammation around a prosthesis, implant or graft in the urinary tract | |
| Infectious complications of procedures, catheters etc. | T83.6* | Infection or inflammation around a prosthesis, implant or graft in the genitals | |
| Infectious complications of procedures, catheters etc. | T84.5* | Infection or inflammation around the joint prosthesis | |
| Infectious complications of procedures, catheters etc. | T84.6* | Infection or inflammation around internal fixation material | |
| Infectious complications of procedures, catheters etc. | T84.7 | Infection or inflammation around other orthopedic prosthesis, implant or graft | |
| Infectious complications of procedures, catheters etc. | T85.7 | Infection or inflammation around other internal prosthesis, implant or graft | |
| Infectious complications of procedures, catheters etc. | T88.0* | Infection after vaccination | |
| Infectious complications of procedures, catheters etc. | T89* | Nosocomial infection | |
| Other infections or sequelae | B98* | Other infectious agents as cause of disease | |
| Other infections or sequelae | B99* | Other or unspecified infectious diseases | |
| Other infections or sequelae | K04.0A | Abscess in dental pulp | |
| Other infections or sequelae | K04.6 | Periapical tooth abscess with fistula | |
| Other infections or sequelae | K04.6A | Alveolar dental abscess with fistula | |
| Other infections or sequelae | K04.7* | Periapical dental abscess without fistula | |
| Other infections or sequelae | K05.2* | Acute periodontitis | |
| 1. Broad category lists are mutually exclusive. | | |  |
| 2. The asterisk (*) denotes that all subcodes are included unless exclusions are noted. Excluded codes are typically included in other categories. | | |  |
| Note that some ICD-10 codes are used only in Denmark. | | |  |

**Supplemental Table 3. Anatomical Therapeutic Chemical (ATC) used to identify community-treated infections**

| **Infection category^1^** | **ATC code^2^** |  |
| --- | --- | --- |
| Antibiotics | J01* including P01AB01 |  |
| Antimycotic (antifungal) | J02* |  |
| Antimycobacterial | J04* |  |
| Antiviral | J05* |  |
| Antiparasitic | P0* (except P01AB01 and P03*) |  |
| 1. Broad category lists are mutually exclusive. | | |
| 2. The asterisk (*) denotes that all subcodes are included unless exclusions are noted. | | |
